# A Hybrid Indoor Ambient Light and Vibration Energy Harvester for Wireless Sensor Nodes

**DOI:** 10.3390/s140508740

**Published:** 2014-05-19

**Authors:** Hua Yu, Qiuqin Yue, Jielin Zhou, Wei Wang

**Affiliations:** 1 College of Optoelectronic Engineering, Chongqing University, Chongqing 400044, China; E-Mail: 20120802065@cqu.edu.cn; 2 Key Laboratory for Optoelectronic Technology & Systems, Ministry of Education of China, Chongqing 400044, China; E-Mail: weiwang8872@gmail.com; 3 National Key Laboratory of Fundamental Science of Micro/Nano-Device and System Technology, Chongqing 400044, China; 4 Department of Electro-Mechanic Engineering, Chongqing College of Electronic Engineering, Chongqing 401331, China; E-Mail: yqq622@163.com

**Keywords:** hybrid energy harvester, indoor ambient light, vibration energy, power conditioning circuit, wireless sensor node

## Abstract

To take advantage of applications where both light and vibration energy are available, a hybrid indoor ambient light and vibration energy harvesting scheme is proposed in this paper. This scheme uses only one power conditioning circuit to condition the combined output power harvested from both energy sources so as to reduce the power dissipation. In order to more accurately predict the instantaneous power harvested from the solar panel, an improved five-parameter model for small-scale solar panel applying in low light illumination is presented. The output voltage is increased by using the MEMS piezoelectric cantilever arrays architecture. It overcomes the disadvantage of traditional MEMS vibration energy harvester with low voltage output. The implementation of the maximum power point tracking (MPPT) for indoor ambient light is implemented using analog discrete components, which improves the whole harvester efficiency significantly compared to the digital signal processor. The output power of the vibration energy harvester is improved by using the impedance matching technique. An efficient mechanism of energy accumulation and bleed-off is also discussed. Experiment results obtained from an amorphous-silicon (a-Si) solar panel of 4.8 × 2.0 cm^2^ and a fabricated piezoelectric MEMS generator of 11 × 12.4 mm^2^ show that the hybrid energy harvester achieves a maximum efficiency around 76.7%.

## 1. Introduction

Periodical battery replacement or recharge has already become one of the major bottlenecks for wireless sensor nodes (WSNs) because of their limited battery life. One possible solution being considered is to harvest energy from various ambient sources, such as light, thermal and vibration energy, supplying power for the WSN [1]. However, these energy sources are not available all the time. In order to harvest energy continuously, it is necessary to design and fabricate a hybrid energy harvester that integrates both solar panels and piezoelectric vibration generators, enabling energy harvesting from light and vibration simultaneously. Harvesting solar energy is a relatively fully fledged technology in outdoor applications with high solar light intensity, but the efficiency of solar panels is very low and output power level is on the order of microwatts under the low light intensity of indoor conditions. Similarly the traditional MEMS-based vibration power generators can only generate power in the microwatt range, and usually have low output voltages on the order of hundreds of mV. There are therefore great challenges in the design of small-scale hybrid indoor ambient light and vibration energy harvesters for wireless sensor nodes, such as accurate light energy prediction, low power MPPT algorithm for low intensity light, and impedance matching of piezoelectric vibration energy harvester.

An accurate and optimized model for predicting the I-V characteristics of small scale solar panels and power scavenging from low light intensity environments is essential to design a small-scale light energy harvesting system. At present, solar panel modelling methods are mainly applied to outdoor high light intensity and big photovoltaic current conditions [2–6]. If applying these models in weak light and small photovoltaic current conditions, they will induce errors between the predicted values and experimental data because the equations were numerically solved with some approximation or simplification [1]. In addition, it is difficult to construct a PSPICE model for circuit simulation that may affect the accuracy of the results [2]. Furthermore, the parasitic series resistance *R_S_* and shunt resistance *R_SH_* of the model were considered as constants during the solution process [3]. Several hybrid energy harvesting approaches were discussed in [6,7], which mainly focused on different power conditioning methods to harvest multiple energy sources. Some researchers have proposed an approach to combine energy harvesting by using an electronic switch or multiplexer to switch between different energy sources, and each energy source is allocated to charge its own energy accumulator. This approach increases the power dissipation of the system [7,8], therefore, designing a high efficiency and ultra-low power consumption power conditioning circuit is critical. The conditioning circuit should extract the maximum energy from the energy sources, accumulate energy and bleed off the energy for meeting the challenges imposed by the instantaneous high power requirements for wireless sensor nodes.

This paper is organized as follows: Section 2 describes the improved five-parameter model of solar panels under the condition of low indoor light intensity. The architecture design and output characteristics of a MEMS vibration energy harvester based on PZT beams array are also introduced. Section 3 discusses a power conditioning circuit for a hybrid energy harvester. Some design considerations about the MPPT algorithm, impedance matching, energy storage, bleed-off, and voltage regulation are also presented in detail. The experimental results are analyzed in Section 4. Finally, Section 5 concludes the paper.

## 2. The Proposed Hybrid Light and Vibration Energy Harvester

### 2.1. The Improved Five-Parameter Model for Solar Panel

**Figure 1.**
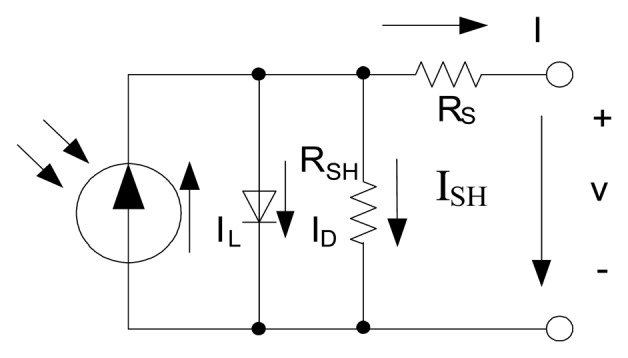
Electrical model of the solar panel.

As shown in Figure 1, the behavior of a solar panel can be modeled with an equivalent circuit, which is composed of a light current source, a forward biased diode, a parasitic series resistance and a shunt resistance.

Under the specific temperature and light illumination conditions, the relationship between output voltage and current can be expressed by Equation (1) [4]:

(1) $$I=I_{L}-I_{O}\left[e^{\frac{V+IR_{S}}{\alpha }}-1\right]-\frac{V+IR_{S}}{R_{SH}}$$

where *I* is the output current, *V* is the output voltage; *I_L_* is the light current, and *I_O_* is the dark saturation current; *α* is the ideality factor, *R_S_* is the series resistance and *R_SH_* is the shunt resistance. The value of five parameters (*I_L_,I_O_,α,R_S_,R_SH_*) vary with the solar panel temperature and light illumination condition. In order to attain I-V curves of the solar panel under different temperature and light intensity, it is necessary to calculate values of the above five parameters.

Firstly, based on the parameters (*V_OC,STC_, I_SC,STC_, V_MPP,STC_, I_MPP,STC_, α_ISC_, β_VOC_*) provided by the manufacturers, the reference values of the above five parameters under the standard test condition (STC) (*I_L,STC_*_,_ *I_O,STC_*_,_ *α_STC_*_,_ *R_S,STC_* and *R_SH,STC_*) are obtained by solving the set of Equations (2)–(6) [4], where *V_OC,STC_* is the open circuit voltage, *I_SC,STC_* is the short circuit current, *I_MPP,STC_* is the current at the maximum power point, and *V_MPP,STC_* is the voltage at the maximum power point. *α_ISC_* and *β_VOC_* are the temperature drift coefficient of the short circuit current and the open circuit voltage, respectively.

For short circuit, *I* = *I_SC,STC_, V* = 0:

(2) $$I_{SC,\text{STC}}=I_{L,\text{STC}}-I_{O,\text{STC}}\left[e^{\frac{I_{SC,\text{STC}}R_{S,\text{STC}}}{\alpha_{\text{STC}}}}-1\right]-\frac{I_{SC,\text{STC}}R_{S,\text{STC}}}{R_{SH,\text{STC}}}$$

For open circuit, *I* = 0, *V* = *V_OC,STC_*:

(3) $$0=I_{L,\text{STC}}-I_{O,\text{STC}}\left[e^{\frac{V_{OC,\text{STC}}}{\alpha_{\text{STC}}}}-1\right]-\frac{V_{OC,\text{STC}}}{R_{SH,\text{STC}}}$$

At the maximum power point, *I* = *I_MPP,STC_, V* = *V_MPP,STC_*:

(4) $$I_{\text{MPP},\text{STC}}=I_{L,\text{STC}}-I_{O,\text{STC}}\left[e^{\frac{V_{\text{MPP},\text{STC}}+I_{\text{MPP},\text{STC}}R_{S,\text{STC}}}{\alpha_{\text{STC}}}}-1\right]-\frac{V_{\text{MPP},\text{STC}}+I_{\text{MPP},\text{STC}}R_{S,\text{STC}}}{R_{SH,\text{STC}}}$$

The derivative of output power with respect to output voltage at the maximum power point is zero. At the maximum power point, ${\frac{dP}{dV}|}_{\text{MPP},\text{STC}}=0$:

(5) $$\frac{I_{\text{MPP},\text{STC}}}{V_{\text{MPP},\text{STC}}}=\frac{I_{O,\text{STC}}}{\alpha_{\text{STC}}}(1-\frac{I_{\text{MPP},\text{STC}}}{V_{\text{MPP},\text{STC}}}\cdot R_{S,\text{STC}})\cdot e^{\left(\frac{V_{\text{MPP},\text{STC}}+I_{\text{MPP},\text{STC}}R_{S,\text{STC}}}{\alpha_{\text{STC}}}\right)}+\frac{R_{SH,\text{STC}}-R_{S,\text{STC}}}{{R_{SH,\text{STC}}}^{2}}$$

The derivative of output current with respect to output voltage at short circuit is reciprocal of the shunt resistance *R_SH_*.

For short circuit, ${\frac{dI}{dV}|}_{I=I_{SC,\text{STC}}}=-\frac{1}{R_{SH,\text{STC}}}$,*I* = *I_SC,STC_, V* =0:

(6) $$\frac{1}{R_{SH,\text{STC}}}=\frac{I_{O,\text{STC}}}{\alpha_{\text{STC}}}\left(1-\frac{R_{S,\text{STC}}}{R_{SH,\text{STC}}}\right)e^{I_{SC,\text{STC}}\frac{R_{S,\text{STC}}}{R_{SH,\text{STC}}}}+\frac{R_{SH,\text{STC}}-R_{S,\text{STC}}}{R_{SH,\text{STC}}^{2}}$$

Secondly, substituting the known parameters (*V_OC,STC_, I_SC,STC_, α_ISC_, β_VOC_*) into the Equations (7) and (8), the short circuit current *I_SC_* and the open circuit voltage *V_OC_* are calculated under specific temperature and light illumination conditions, respectively [6].

(7) $$I_{SC}=I_{SC,\text{STC}}\frac{G}{G_{\text{STC}}}+\alpha_{I_{SC}}\cdot I_{SC,\text{STC}}\cdot (T_{C}-T_{\text{STC}})$$

(8) VOC=VOC,STC+α⋅STCln[GGSTC]+βVOC⋅VOC,STC⋅(TC−TSTC)

where *G* is the solar irradiance, *G_STC_* is the solar irradiance under the STC, *T* is the solar panel working temperature, and *T_STC_* is the solar panel working temperature under the STC.

According to the Equations (2), (3) and (6), the following Equations (9)–(11) are valid:

(9) $$\begin{array}{cc} I_{SC}=I_{L}-I_{O}\left[e^{\frac{I_{SC}R_{S}}{\alpha }}-1\right]-\frac{I_{SC}R_{S}}{R_{SH}} & \left(I=I_{SC},V=0\right) \end{array}$$

(10) $$\begin{array}{cc} 0=I_{L}-I_{O}\left[e^{\frac{V_{OC}}{\alpha }}-1\right]-\frac{V_{OC}}{R_{SH}} & \left(I=0,V=V_{OC}\right) \end{array}$$

(11) $$\begin{array}{cc} \frac{1}{R_{SH}}=\frac{I_{O}}{\alpha }\left(1-\frac{R_{S}}{R_{SH}}\right)e^{I_{SC}\frac{R_{S}}{R_{SH}}}+\frac{R_{SH}-R_{S}}{R_{SH}^{2}} & \left({\frac{dI}{dV}|}_{I=I_{SC}}=-\frac{1}{R_{SH}},I=I_{SC},V=0\right) \end{array}$$

As the equivalent series resistance *R_S_* and the equivalent shunt resistance *R_SH_* of the traditional model are usually dependent on the manufacturers' technologies, the terms *R_S_* and *R_SH_* are considered as constants for the traditional solar panel model. In order to predict the output characteristic curves of the solar panel more accurately under indoor low light intensity conditions, the parameters *R_S_* and *R_SH_* are amended in the proposed improved model by using Equations (12) and (13). The amended parameters *R_S_* and *R_SH_* now become functions of temperature and light intensity. This optimized model improves the accuracy of light energy prediction by amending the expressions of *R_S_* and *R_SH_*.

(12) $$R_{S}=\frac{R_{S,\text{STC}}\cdot \left[\frac{V_{OC}}{\alpha }-1\right]+R_{SH,\text{STC}}\cdot \left[1-\frac{I_{SC}R_{S,\text{STC}}}{\alpha }\right]}{\frac{V_{OC}-I_{SC}R_{SH,\text{STC}}}{\alpha }}$$

(13) $$R_{SH}=R_{SH,\text{STC}}-R_{S}$$

Thirdly, the five parameters (*I_L_, I_O_, α, R_S_*, and *R_SH_*) are obtained by solving the equation set (9)–(13) [5] based on the calculated parameters *I_SC_* and *V_OC_*.

Once the five parameters have been calculated, the I-V curves of the solar panel under different temperature and light intensity can be obtained. The prediction accuracy of the model is verified by comparing the simulation results with experimental results under various light illumination conditions.

According to previous research, amorphous-silicon (a-Si) solar panel is particularly well suited for application in indoor low light intensity conditions because its density matches the spectral power density of indoor light (fluorescent or LED) very well [9,10]. Therefore, a-Si solar panel is used in the experiments. The technical characteristics of the a-Si solar panel are shown in Table 1.

**Figure 2.**
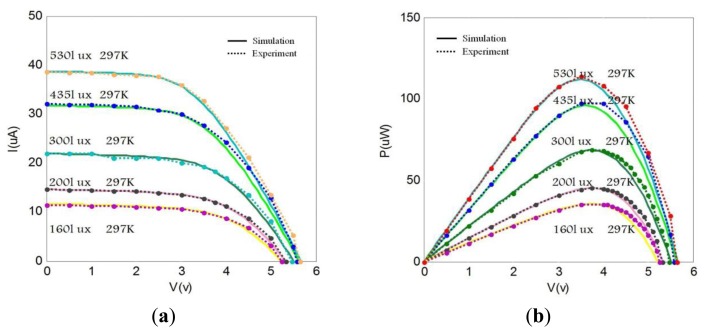
Performance evaluation of the improved model. (**a**) Comparison of the I-V simulation with experiment curves. (**b**) Comparison of the P-V simulation with experiment curves.

**Figure 3.**
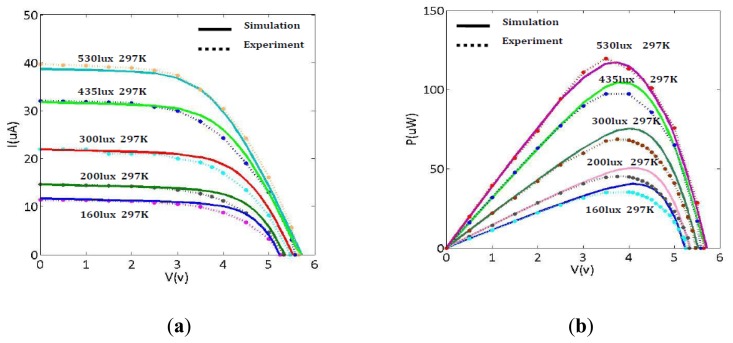
Performance evaluation of the traditional model. (**a**) Comparison of the I-V simulation curves and the I-V experiment curves. (**b**) Comparison of the P-V simulation curves and the P-V experiment curves.

Comparing the I-V or P-V curves shown in Figures 2 and 3, the improved model shows better matching between simulation and experimental results under indoor light illumination conditions. Analysis shows the improved five-parameter model has higher prediction accuracy than the traditional modeling method under the same light irradiance conditions. Specifically, the discrepancy between simulation and experiment results shown in Figure 3 is larger than that in Figure 2. Moreover, this discrepancy becomes even larger with the decrease of light irradiance in the traditional model. For example, the relative error of maximum power is up to 2.74% at 530 lux for the traditional model, while the relative error of maximum power is 0.91% at 530 lux for the improved model. It's clear that the relative error of maximum power is increased to 15% at 160 lux for the traditional model, while the relative error of maximum power is only increased 1.05% at 160 lux for the improved model. Thus, conclusion is drawn that the improved modeling method possesses higher predictive accuracy than the traditional modeling method for lower illumination conditions (for example, at 160–200 lux). These errors have been caused by not taking into account the effects of parasitic series resistance *R_S_* and shunt resistance *R_SH_* on output characteristic of solar cell, in the traditional five-parameter model which considering *R_S_* and *R_SH_* as constants [3].

The parameters *R_S_* and *R_SH_* are amended in the proposed improved model. The amended parameters are functions of temperature and light intensity, so the proposed model improves the prediction accuracy. In addition, *I_L_* is assumed to be linearly proportional to the solar irradiance. The simultaneous solution of the equations is performed by using a non-linear equation solver. This method of obtaining the model parameters is usually inadequate because of the difficulties for calculations. It introduces errors during the solving process of the traditional modeling method.

### 2.2. Architecture Design and Output Characteristics of Vibration Energy Harvester

**Figure 4.**
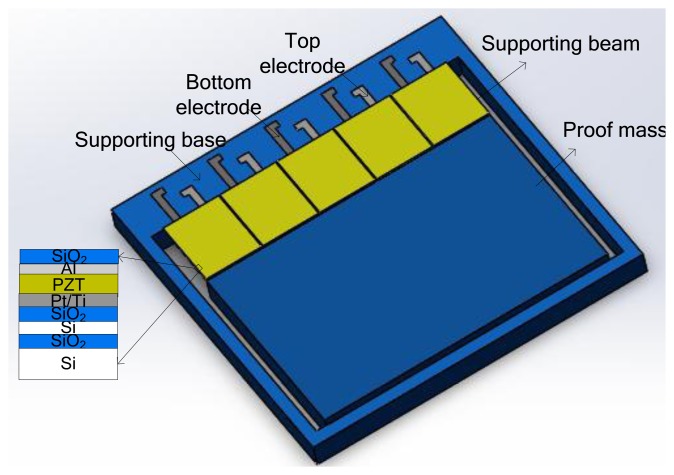
Schematic of MEMS vibration energy harvester.

The MEMS vibration energy harvester is composed of a five PZT cantilevers array which is integrated with a large Si proof mass. The PZT thin film layers are sandwiched between the top electrode and the bottom electrode which are constructed on silicon supporting beams. Five PZT beams are electrically isolated with each other. Each of the top Al electrode and the bottom Pt/Ti electrode are connected to bonding pads individually. Figure 4 shows the schematic of the proposed MEMS vibration energy harvester.

**Figure 5.**
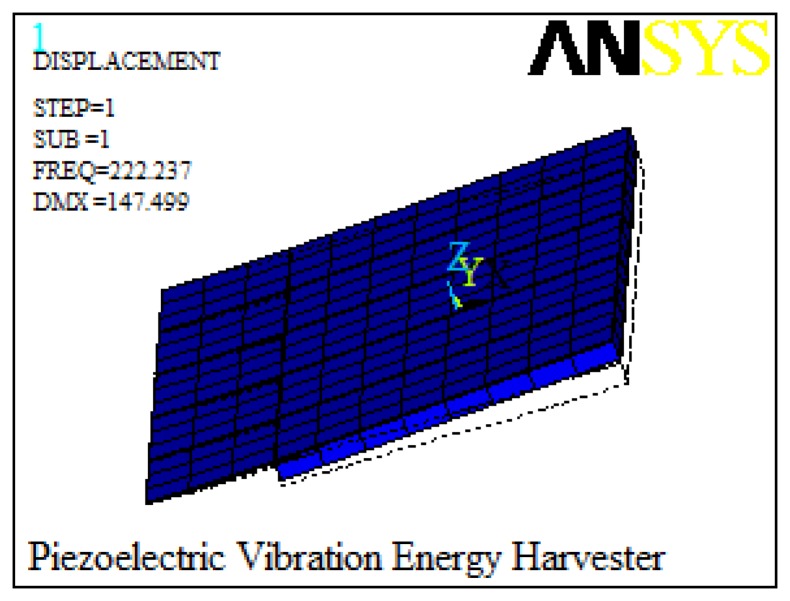
The first resonant frequency of MEMS vibration energy harvester.

When the bending results in a strain distributed along the beam, each PZT element vibrates in phase, which generates electrical energy based on the transverse mode (d31) [11,12]. The modal analysis simulated by ANSYS is used to calculate the resonant frequency of the harvester. The first resonance frequency is 222.2 Hz, as shown in Figure 5.

**Figure 6.**
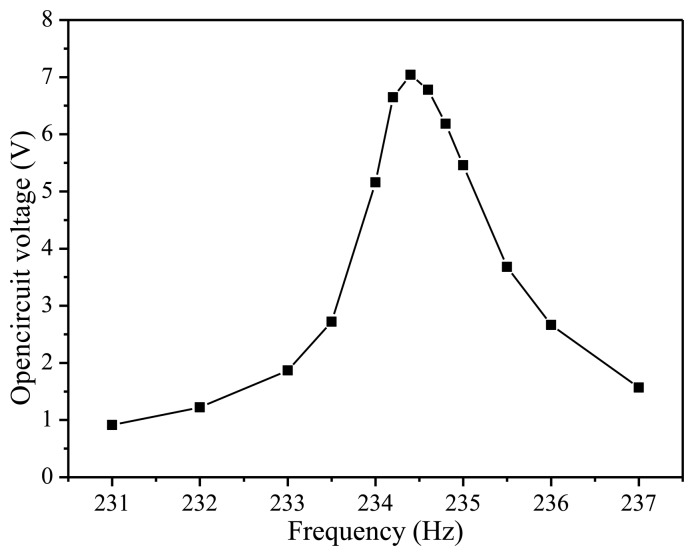
The output voltage characteristic of MEMS vibration energy harvester.

**Figure 7.**
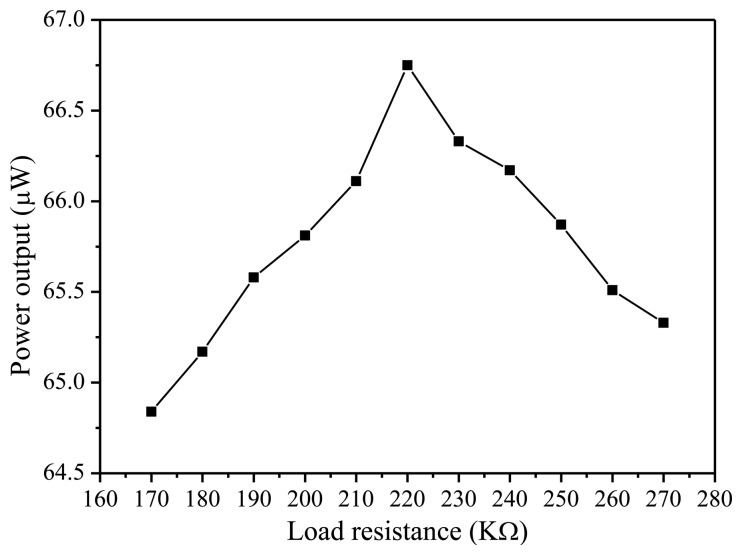
The output power characteristic of MEMS vibration energy harvester.

Traditionally, a MEMS piezoelectric energy harvester has the issue of low voltage output which limits its application. In this paper, in order to achieve the high output voltage, the five PZT beams are connected in series. The output characteristics of this MEMS piezoelectric energy harvester are measured experimentally. The output voltage of five serially connected beams is shown in Figure 6 with the frequency varying from 231 Hz to 237 Hz at 5 m/s^2^ vibration acceleration. The experiment results show that the maximum open circuit voltage is 7.04 V at the resonant frequency of 234.5 Hz. The error between the simulated and measured resonant frequency is about 4.2%. Figure 7 shows the average output power characteristic versus loads at resonant frequency. It is obvious that the maximum output power of 66.75 μW is obtained at 5 m/s^2^ acceleration with an optimal resistive load of 220 kΩ.

## 3. The Power Conditioning Circuit

**Figure 8.**
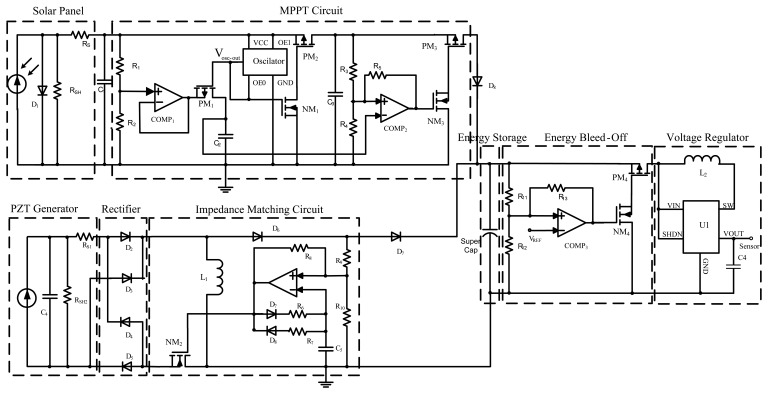
The schematic of the proposed power conditioning circuit.

As shown in Figure 8, the proposed power conditioning circuit consists of a MPPT circuit for ambient light energy, a AC-DC rectifying circuit and impedance matching circuit for vibration energy, energy storage, energy bleed-off and voltage regulator. The basic idea is to scavenge the energy from two energy sources as much as possible, store the energy in a single super-capacitor and discharge it when it is enough to supply the load for an established amount of time. The quiescent current consumption of the power conditioning circuit should be as small as possible. In order to achieve power from the solar panel and MEMS vibration energy harvester, the MPPT circuit is implemented by using fractional open circuit voltage algorithm and the impedance matching circuit is designed by using the DC-DC buck-boost converter, respectively. Moreover, the MPPT circuit is autonomously controlled by purely analog circuit, without the use of a traditional digital micro-controller, which dramatically decreases the power dissipation of the circuit. The different parts of the circuit are analyzed with detailed information as follows.

### 3.1. MPPT Circuit

The power level obtainable from small-scale indoor light energy harvesting system usually varies in the μW range. It is fairly challenging to extract maximum power from low light intensity environment. Among several conventional MPPT algorithms, the fractional open circuit voltage method is based on the observation that the ratio of solar cell's MPP voltage *V_MPP_* to its open circuit voltage *V_OC_* is nearly constant (*V_MPP_* ≈ *K*_1_*V_OC_*). According to previous researches, the coefficient *K_1_* is a constant in the range of 0.7 to 0.8. Once the constant *K_1_* is known, *V_MPP_* is obtained by measuring *V_OC_* periodically. The implementation of this MPPT algorithm is simple and inexpensive [13–17]. Moreover, it consumes less power compared with other MPPT methods. Therefore, the special MPPT circuit by using the fractional open circuit voltage algorithm is used in the energy harvesting device.

This MPPT circuit consists of a sample and hold circuit, an oscillator, a control switch and a voltage comparator with hysteresis. The sample and hold circuit is composed of two resistors, a voltage follower and a sample capacitor. The resistors are used to divide the open circuit voltage *V_OC_* to the required *V_MPP_*. The sample capacitor *C_2_* is charged by the sampling value of *V_OC_*. In order to sample the input signal, the switch *PM_1_* connects the capacitor with the output of the buffer amplifier. The buffer amplifier outputs voltage to charge the capacitor *C_2_* so that the voltage across the capacitor is proportional to the open circuit voltage *V_OC_*. In hold mode, the switch *PM_1_* disconnects the capacitor from the buffer by using the sample pulse signal which is produced by the oscillator circuit. If the value of coefficient *K_1_* is assumed to be 0.75 (*V_C3_* = 0.75*V_OC_*), according to the parameters of solar panel and the above fractional open circuit voltage method, the solar panel is working at the maximum power point. Based on the schematic of circuit in Figure 8, the Equation (14) is valid. So the super-capacitor is charged by the maximum power output from the solar panel, which is implemented by using the MPPT circuit when Equation (15) holds.

(14) $$\frac{V_{OC}R_{2}}{R_{1}+R_{2}}=\frac{V_{C3}R_{4}}{R_{3}+R_{4}}$$

(15) $$\frac{R_{2}}{R_{1}+R_{2}}=\frac{0.75R_{4}}{R_{3}+R_{4}}$$

**Figure 9.**
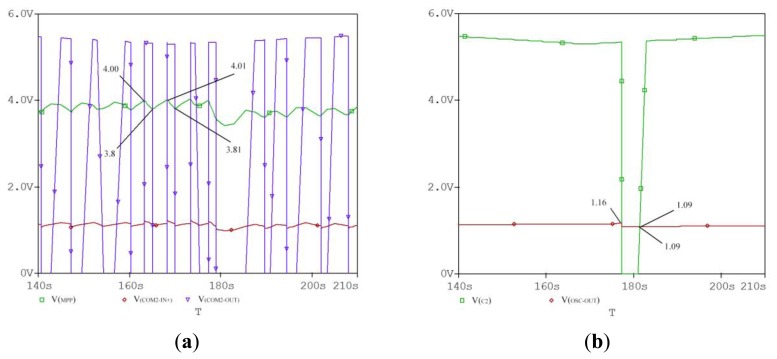
The simulated voltage curves of MPPT circuit under the specific condition (G = 200 lux, T = 297 K). (**a**) The curves of *V_MPP_, V_COM2-OUT_*, and *V_COM2-IN_*_+_. (**b**) The curves of *V_OSC-OUT_* and *V_C2_*.

Figure 9 shows the simulated output voltage curves of MPPT circuit. It is clearly shown that the maximum power point voltage is 3.8–4.0 V, which is approximately equal to the measured value of *V_MPP_*. Moreover, the output voltage *COMP_2_* with hysteresis becomes high and turns the switch *PM_3_* on when the voltage is equal to the *V_MPP_*. Therefore, the super-capacitor is charged by the maximum power point voltage *V_MPP_*. The charging efficiency of the super-capacitor has been greatly increased by the aforementioned mechanism. In addition, the MPPT circuit is implemented by using analog discrete components, which significantly improves the whole harvester efficiency compared with using digital signal processor.

### 3.2. Impedance Matching Circuit

As the output voltage of the vibration energy harvester is an AC voltage signal, adopting a full wave bridge rectifier to convert the signal to DC voltage is needed. The bridge rectifier uses four individual rectifying diodes connected in a closed loop “bridge” configuration to produce the desired output. The main advantage of this bridge circuit is that it does not require a special center tapped transformer, thereby reducing its size and power dissipation.

Considering the nature of high output impedance characteristics of PZT vibration energy harvester, the impedance matching circuit must be adopted in order to achieve maximum output power. The effective input resistance of the discontinuous conduction mode (DCM) DC-DC buck-boost converter is expressed by Expression (16) [18]:

(16) $$R_{IN}=\frac{V_{\text{RECT}}}{\frac{1}{T_{S}}\int_{0}^{DT_{S}}I_{L}dt}=\frac{V_{\text{RECT}}}{\frac{1}{T_{S}}\int_{0}^{DT_{S}}\frac{V_{\text{RECT}}}{L}dt}=\frac{2Lf_{s}}{D^{2}}$$

where *L* is the used inductance, *f_S_* is switching frequency, *D* is duty cycle of the transistor *Q*_1_. Expression(16) shows the input resistance *R_IN_* is controlled by the duty cycle of the switching element. For fixed values of the duty cycle and the switching frequency, the DCM buck-boost converter without an input filter capacitor behaves as a lossless resistor to match the source impedance of the piezoelectric energy harvester, thus harvesting the maximum power from the energy source. The low power comparator with an *RC* network is connected acting as a simple oscillator, which is used to generate the pulse width modulation (PWM) signal for turning the power switch *NM*_2_ on and off. The duty cycle and switching frequency can be adjusted by choosing appropriate *R_6_, R_7_* and *C_5_* to achieve the maximum power [19].

### 3.3. Energy Storage and Bleed-Off Circuit

The harvested energy should be accumulated in an energy storage element until it is enough to supply the load for an established amount of time. There are two options, either using a battery or a super-capacitor. A proper capacitance of super-capacitor is chosen as an energy storage element by comparing their performance. The super-capacitor should be disconnected from the load during the energy accumulation stage to prevent energy leakage to the load. The super-capacitor is connected to the load only if the accumulated energy is large enough to drive it. This means that the accumulated energy should be monitored all the time. The voltage comparator with hysteresis is connected to monitor the super-capacitor's voltage and control the super-capacitor's charging and discharging process.

### 3.4. Output Voltage Regulator and Wireless Sensor Node Load

In order to supply a stable voltage for the wireless sensor node load, a commercial low power DC-DC voltage regulator is adopted as the output unit providing power supply to the wireless sensor node. In order to improve the efficiency of the voltage regulator, the start-up enable signal for DC-DC is continuously monitored. The voltage regulator begins to work only if the accumulated energy is large enough, which improves the efficiency of the system. The SmartNode WTS-G0 is a high-accuracy digital wireless temperature sensor, and its rated operation voltage is 3.6 V. The operation modes of the sensor node include sleeping, sensing, receiving and transmitting (peak). Extremely low sleep current (2.5 μA) and sensing current (7 μA) combined with periodic and timed wake-up is used to minimize the average power consumption. It works in the so-called burst mode. More power (50 mW@17 dbm) is needed for wireless sensor node working in transmitting/receiving mode than other working modes. However, it transmits and receives information very fast in very small time slots (5 ms).

## 4. Experimental Results

**Figure 10.**
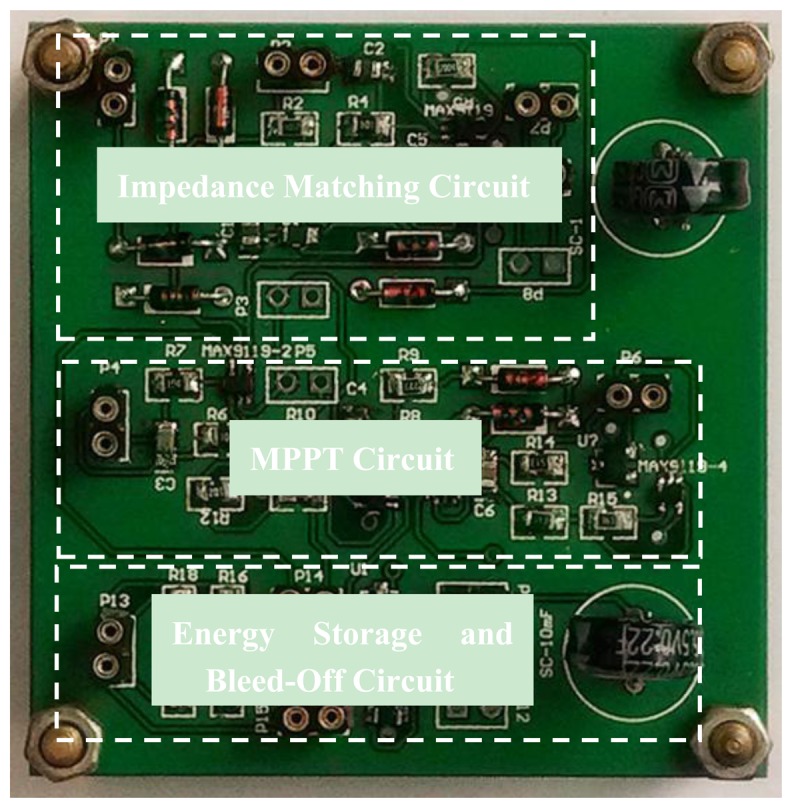
The photo of the power conditioning circuit.

As shown in Figure 10, a photo of power conditioning circuit for the indoor ambient light and vibration hybrid energy harvester for wireless sensor node shows an impedance matching circuit for the vibration energy harvester, MPPT circuit for light energy, energy storage and bleed-off circuit. In order to evaluate the performance of the circuit, the charging-discharging processes of a super-capacitor and the duty cycle of a wireless sensor are analyzed, respectively. The quantitative analysis of energy loss is performed.

**Figure 11.**
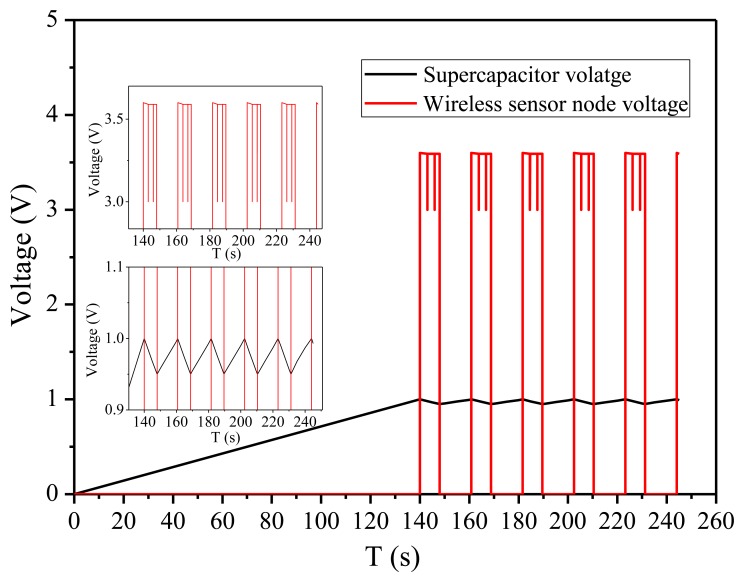
The voltage across the storage super-capacitor and wireless sensor node.

As shown in Figure 11, the energy bleed-off circuit and DC-DC voltage regulator will start up as soon as the voltage across the super-capacitor is charged to 1.0 V. The initial charge time is 140 s for the super-capacitor's voltage charged from 0 V to 1.0 V. The super-capacitor's voltage will begin to drop when its energy is discharged to the load. Once the voltage across the super-capacitor drops below 0.95 V, the DC-DC voltage regulator will shut off. The discharging time is 8 s. The DC-DC voltage regulator will start up again when the super-capacitor voltage is charged to 1.0 V next time. The charging time is 12.8 s for the super-capacitor's voltage charged from 0.95 V to 1.0 V. The working cycle time of the wireless temperature sensor is configured to 3 s in the experiment. The bleed-off energy of super-capacitor during one operating cycle is calculated as follows:

(17) $$E_{sc}=\frac{1}{2}{{CV}_{\text{THR}}}^{2}-\frac{1}{2}{{CV}_{\text{THF}}}^{2}=\frac{1}{2}\times 0.022\times (1.0^{2}-0.95^{2})=1.1mJ$$

The energy consumed by the wireless temperature sensor node at one operating cycle time is calculated as follows:

(18) $$E_{\text{load}}=PT=P_{1}T_{1}+P_{2}T_{2}=0.025mW\times 2.995s+50mW\times 0.005s=0.325mJ$$

The experiment showed that the proposed energy harvesting system can successfully drive the wireless sensor node to work well for 2–3 duty cycles. The maximum power point voltage and current of solar panel are multiplied to calculate the input power. Similarly, it is easy to calculate the energy stored in the super-capacitor. The efficiency of the whole energy harvesting system under the specific indoor light illumination (G = 200 lux, T = 297 K) and vibration frequency of 234.5 Hz condition is as follows:

(19) $$\eta =\frac{P_{SC}}{P_{IN}}=\frac{E_{SC}/T}{P_{\text{MPP}\_S}+P_{\text{MPP}\_V}}=\frac{\frac{1}{2}\frac{C}{T}({V^{2}}_{\text{THR}}-{V^{2}}_{\text{THF}})}{V_{\text{MPP}\_S}I_{\text{MPP}\_S}+V_{\text{MPP}\_V}^{2}/R}=\frac{\frac{1.1mJ}{12.8s}}{45.38\mu W+66.75\mu W}=76.7\%$$

Although the system works at the maximum power point of solar panel and resonant frequency of vibration, because of the losses at the switch device, comparator, DC-DC voltage regulator, and the super-capacitor, the efficiency of system can still be increased by improving the circuit architecture and adopting an integrated circuit with ultra-low power dissipation in future research work.

## 5. Conclusions

A hybrid indoor ambient light and vibration energy harvesting scheme that uses only one power conditioning circuit for the combined output power harvested from both energy sources is proposed in this paper. It reduces the number of components and lessens the power loss. An improved five-parameter model for solar panels is presented for indoor low intensity light conditions, which is proven to be more accurate for predicting the harvesting energy than the traditional model. A MEMS piezoelectric cantilever arrays generator is proposed to increase the output voltage. The MPPT for the small-scale solar panel is implemented by using analogy circuit components, which improves the whole harvester efficiency compared to using a digital signal processor. The optimal impedance matching technique of the vibration energy harvester is implemented by using a DCM DC-DC buck-boost converter. An efficient mechanism of energy accumulation and bleed-off is also discussed. The experimental results show that the proposed hybrid energy harvesting system can meet the voltage, current, and power consumption needs of a wireless sensor node under indoor low intensity light and resonant vibration conditions. This work provides a more promising and complete power supply solution for wireless sensor nodes, which is suitable for applications that require self-powered, renewable, maintenance-free, and intermittent power sources in indoor environments where both light and vibration energy are available.

## Figures and Tables

**Table 1.** Technical parameters of a-Si solar panel (G = 200 lux; T = 297 K).

**Symbol**	**Parameter**	**Value**
*A*	Surface Area	9.6 cm^2^
*V_OC_* (200 lux)	Open Circuit Voltage	5.33 V
*I_SC_*	Short Circuit Current	14.6 μA
*V_MPP_*	MPPT Voltage	3.75 V
*I_MPP_*	MPPT Current	12.1 μA
